# Small molecule multitarget antiangiogenic inhibitor treatments for advanced thymic epithelial tumors: A retrospective study

**DOI:** 10.1111/1759-7714.15167

**Published:** 2023-11-27

**Authors:** Wanji Shen, Ying Jin, Ying Yu, Ning Chen, Yun Fan

**Affiliations:** ^1^ Postgraduate Training Base Alliance of Wenzhou Medical University Wenzhou China; ^2^ Zhejiang Cancer Hospital, Hangzhou Institute of Medicine (HIM) Chinese Academy of Sciences Hangzhou China; ^3^ Department of Oncology The Second Clinical Medical College, Zhejiang Chinese Medical University Hangzhou China

**Keywords:** small molecule multitarget antiangiogenic inhibitors, thymic epithelial tumor, thymic carcinoma, thymoma

## Abstract

**Background:**

Thymic epithelial tumors (TETs) are rare malignant tumors with limited treatment options. No established second‐line treatment regimen is available following the preferred first‐line chemotherapy, resulting in unsatisfactory efficacy and poor prognosis for patients with advanced TETs. This study aimed to evaluate the efficacy of small molecule multitarget antiangiogenic inhibitors as well as the prognostic factors for advanced TETs.

**Methods:**

A retrospective study was conducted using data from a real‐world database. Clinical information and survival follow‐up data were collected from 52 patients with advanced TETs who received small molecule multitarget antiangiogenic inhibitors at Zhejiang Cancer Hospital between August 10, 2016 and August 10, 2022. The short‐term efficacy of the treatments, survival time of the patients, and relevant prognostic factors of advanced TETs were analyzed.

**Results:**

Out of the 52 patients included in this study, 16 had thymoma and 36 had thymic carcinoma. The 52 patients had an overall response rate of 21.1% and a disease control rate of 94.2%. In addition, the median progression‐free survival (PFS) was 8.05 months, and the overall survival (OS) was 25.00 months. Apatinib was given to 33 patients, anlotinib to 15 patients, and sunitinib or lenvatinib to four patients. Only seven patients received antiangiogenic inhibitors as their first‐line therapy, 27 patients as their second‐line therapy, and 18 patients as third‐line or subsequent therapy. Meanwhile, 42 patients received monotherapy with an antiangiogenesis inhibitor, while 10 patients received combination therapy. Univariate analysis indicated that the combined treatment was associated with a superior OS (*p* = 0.044); multivariate analysis indicated that the combined treatment was an independent prognostic factor for PFS (*p* = 0.014) and OS (*p* = 0.012).

**Conclusion:**

The findings suggest that small molecule multitarget antiangiogenic inhibitors are efficacious as second or post‐line treatments as a viable alternative treatment option for patients with advanced TETs.

## Graphical abstract

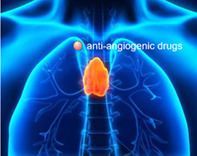

## INTRODUCTION

Thymic epithelial tumors (TETs) are rare tumors that occur in the anterior mediastinum, accounting for less than 1% of adult malignancies.^1^, ^2^, ^3^ TETs include thymoma and thymic carcinoma and can be further classified into A, AB, B1, B2, B3, and C (i.e., thymic carcinoma) subtypes based on histological characteristics.^4^, ^5^ All subtypes are associated with recurrence and metastasis, while the invasiveness of TETs increases with their histological subtype. Known prognostic indicators for TETs include histological type, stage, and the possibility of surgical resection.^6^ Surgical resection is the recommended treatment for early‐stage TETs. However, anthracycline or platinum‐based chemotherapy and/or radiation is the main first‐line therapy for advanced unresectable TETs.^7^ Unfortunately, advanced TETs are associated with a poor prognosis. In previous studies, patients with thymomas were found to have a median progression‐free survival (mPFS) of 12.10–21.00 months and an objective response rate (ORR) of 15.0%–40.0%. Patients with thymic carcinomas, on the other hand, had a mPFS of 2.90–4.00 months and an ORR of 5.0%–26.0%.^8^, ^9^ Currently, there is no established second‐line treatment for TETs.

Tumor angiogenesis is an important therapeutic target for malignant tumors. Angiogenesis, the formation of new blood vessels, plays a critical role in the development of TETs. Previous studies revealed high expression levels of vascular endothelial growth factor (VEGF)‐A and its receptors VEGFR1 and VEGFR2 in TETs. Furthermore, the density of microvessels and expression level of VEGF have been linked to the invasion, infiltration, and clinical staging of TETs.^10^ Several clinical studies have explored the efficacy of targeted therapies in TETs. In a single‐arm phase II clinical study, 41 patients with TETs who had previously failed chemotherapy were administered oral sunitinib; the patients with thymoma (*n* = 16) had an ORR of 6.0% and mPFS of 8.50 months, while the patients with thymic carcinoma (*n* = 25) had an ORR of 26.0% and mPFS of 7.20 months.^11^ Another single‐arm phase II clinical study evaluated the efficacy of lenvatinib in 42 patients with advanced thymic carcinoma, achieving an ORR of 38.0% and mPFS of 9.30 months.^12^ Furthermore, a single‐arm phase II screening study was conducted on 19 patients with TETs who had previously failed chemotherapy; the treatment with regorafenib resulted in mPFS of 9.60 months and an OS of 33.80 months.^13^ Apatinib, an oral tyrosine kinase inhibitor, was also evaluated in a single‐arm phase II clinical trial including 25 patients who had metastatic or relapsed TETs; an ORR of 40.0% and mPFS of 9.00 months were found in these patients.^14^ In a retrospective analysis of 20 patients with advanced TETs who had failed platinum‐containing chemotherapy, daily continuous sunitinib treatment achieved an ORR of 31.6% and an overall mPFS of 7.30 months among 19 patients with evaluable response; for patients with thymoma, the mPFS was 7.30 months and ORR was 41.0%; for patients with thymic carcinoma, the mPFS was 6.80 months and ORR was 41.0%.^15^ Another retrospective study was conducted in China, which included 22 patients who received anlotinib monotherapy or anlotinib combined with chemotherapy/immunotherapy; the study reported an ORR of 9.1%, mPFS of 12.00 months, and OS of 24.00 months.^16^

These previous findings emphasize the potential effectiveness of targeted therapies on TETs. However, these studies were mostly single‐center, single‐arm studies based on a small number of cases due to the relatively low incidence of TETs. Meanwhile, the efficacy of antiangiogenic inhibitors in the Chinese TET population has not been widely reported. Therefore, we utilized a real‐world database to analyze the efficacy and safety of multitarget antivascular inhibitors in managing advanced TETs. Additionally, we also investigated the clinical factors that have impact on survival projections of the patients.

## METHODS

### Study design and data collection

**FIGURE 1:**
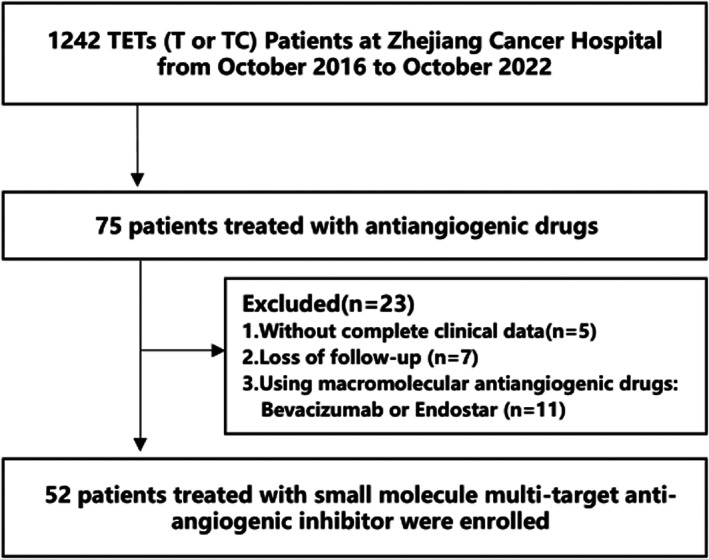
Study flow chart.

A total of 1242 patients with pathologically documented TETs at Zhejiang Cancer Hospital from October 2016 to October 2022 were screened, among which 52 patients treated with small molecule multitarget antiangiogenic inhibitors were enrolled. The major inclusion criteria were: Eastern Corporation Oncology Group performance status (ECOG PS) of 0–2; stage IVA or IVB defined by the Masaoka‐Koga classification; at least one measurable lesion defined by the Response Evaluation Criteria in Solid Tumors (RECIST) version 1.1; with complete clinic data and follow‐up information; treated with small molecule multitarget antiangiogenic inhibitors. The major exclusion criteria were malignant tumors in other sites (Figure 1). Ethical approval for conducting this study was obtained from the Institutional Ethics Committee at Zhejiang Cancer Hospital (no. IRB‐2023‐548). As a retrospective study, individual patient consent was not required.

### Efficacy assessment

PFS refers to the time between initiation of therapy and the onset of disease progression, or death from any cause, whichever is sooner. OS refers to the time from the start of therapy to death from any cause. Disease progression is primarily assessed by clinicians on imaging. As defined by the investigators, ORR refers to the proportion of patients with a complete response (CR) or partial response (PR). The disease control rate (DCR) is the percentage of patients with CR, PR, or stable disease (SD).

### Statistical analysis

GraphPad Prism (version 9.0.0) and SPSS (version 24.0) were used for statistical analysis. A chi squared test was performed for features with normally distributed distributions. For PFS and OS survival analyses, Kaplan–Meier methods were applied. In order to obtain adjusted odds ratio (OR) and 95% confidence intervals (95% CI), univariate and multivariate binary logistic regressions were conducted. The significance level was set at *p* < 0.05.

## RESULTS

### Characteristics of patients

A total of 52 patients with TETs who had been treated with small molecule multitarget antiangiogenic inhibitors were enrolled in this retrospective study (Figure 1). The baseline characteristics of all the patients are shown in Table 1. These patients had a median age of 55 (range: 35–82) years. A total of 69.2% of patients (*n* = 36) were under 60 years of age, 55.8% (*n* = 29) were male, 57.7% (*n* = 30) were current or former smokers, 92.3% (*n* = 48) had an ECOG PS of 0–1, and 88.5% (*n* = 46) had a Masaoka‐Koga staging of IVB. The histology of the patients was 16 (30.8%) thymoma and 36 (69.2%) thymic carcinoma. The most common type of thymoma was type B2 (*n* = 7), and the most common type of thymic carcinoma was squamous cell carcinoma (*n* = 22). Only one thymoma patient had myasthenia gravis. The most common sites of distant metastases were lung (69.2%, 36/52) and liver (32.7%, 17/52). Moreover, 82.7% (*n* = 43) patients had ≥2 metastatic lesions. Furthermore, of the 52 patients, 25 (48.1%) had undergone surgery (*p* < 0.001), and 41 (78.8%) had received radiotherapy. An ultrasound‐ and computed tomography‐guided needle biopsy or thoracoscopic examination is commonly performed as a supplementary histopathological examination in patients with TETs who are not undergoing surgery. Seven (13.5%,7/52) patients received antiangiogenic therapy as their first‐line treatment, while the median number of prior treatment lines was two lines (range: 0–5). The majority of patients (80.8%, 42/52) received antiangiogenic inhibitors as monotherapy. Among the patients who received combined therapy, the major (90.0%, 9/10) histology of TETs was thymic carcinoma, seven (70.0%, 7/10) patients received immune checkpoint inhibitors as combined drugs, and three thymic carcinoma patients received chemotherapy combined with antiangiogenesis therapy. Among all the patients treated with small molecule multitarget antiangiogenic drugs, 33 (63.5%) were treated with apatinib, 15 (28.8%) with anlotinib, and four (7.7%) with lenvatinib or sunitinib. No statistically significant differences in age, gender, smoking, ECOG PS, staging, myasthenia gravis, site or number of metastatic lesions, radiotherapy, lines of antiangiogenesis therapy, single or combined regimens, or the type of the antiangiogenesis drugs were found between the two groups.

**TABLE 1:** Characteristics of patients with thymic epithelial tumor.

Characteristics	Total (*N* = 52)	Thymoma (*N* = 16)	Thymic carcinoma (*N* = 36)	*p*‐value
Median age (years)	55 (35–82)	53 (38–82)	56 (35–73)	0.354
<60, *n* (%)	36 (69.2%)	13 (81.3%)	23 (63.9%)	
≥60, *n* (%)	16 (30.8%)	3 (18.7%)	13 (36.1%)	
Gender, *n* (%)				0.515
Male	29 (55.8%)	10 (62.5%)	19 (52.8%)	
Female	23 (44.2%)	6 (37.5%)	17 (47.2%)	
Smoking history, *n* (%)				0.640
Yes	30 (57.7%)	10 (62.5%)	20 (55.6%)	
No	22 (42.3%)	6 (37.5%)	16 (44.4%)	
ECOG PS, *n* (%)				0.761
0–1	48 (92.3%)	14 (87.5%)	34 (94.4%)	
2	4 (7.7%)	2 (12.5%)	2 (5.6%)	
Masaoka‐Koga staging, *n* (%)				0.539
IVA	6 (11.5%)	3 (18.7%)	3 (8.4%)	
IVB	46 (88.5%)	13 (81.3%)	33 (91.6%)	
Histological type, *n* (%)				
Thymoma	16 (30.8%)	‐	‐	
*A*	1 (1.9%)	1 (6.2%)	‐	
*AB*	2 (3.8%)	2 (12.5%)	‐	
*B1*	0 (0.0%)	0 (0.0%)	‐	
*B2*	7 (13.5%)	7 (43.8%)	‐	
*B3*	6 (11.5%)	6 (37.5%)	‐	
Thymic carcinoma	36 (69.2%)	‐	‐	
Squamous cell carcinoma, NOS	22 (42.3%)	‐	22 (61.1%)	
Neuroendocrine	3 (5.8%)	‐	3 (8.4%)	
Uncategorized	11 (21.2%)	‐	11 (30.5%)	
Liver metastases, *n* (%)				0.268
Yes	17 (32.7%)	3 (18.7%)	14 (38.9%)	
No	35 (67.3%)	13 (81.3%)	22 (61.1%)	
Number of metastatic lesions, *n* (%)				1.000
1	9 (17.3%)	3 (18.7%)	6 (16.7%)	
≥2	43 (82.7%)	13 (81.3%)	30 (83.3%)	
Surgical operation history, *n* (%)				<0.001
Yes	25 (48.1%)	14 (87.5%)	11 (30.6%)	
No	27 (51.9%)	2 (12.5%)	25 (69.4%)	
Radiotherapy history, *n* (%)				0.747
Yes	41 (78.8%)	11 (68.8%)	30 (83.3%)	
No	11 (21.2%)	5 (31.2%)	6 (16.7%)	
Prior therapy lines, *n* (%)				0.771
0–1	34 (65.4%)	10 (62.5%)	24 (66.7%)	
≥2	18 (34.6%)	6 (37.5%)	12 (33.3%)	
Antiangiogenesis therapy, *n* (%)				0.229
Single drug	42 (80.8%)	15 (93.8%)	27 (75.0%)	
Combined treatment	10 (19.2%)	1 (6.2%)	9 (25.0%)	
Antiangiogenesis drugs, *n* (%)				0.382
Apatinib	33 (63.5%)	11 (68.8%)	22 (61.1%)	
Anlotinib	15 (28.8%)	5 (31.2%)	10 (27.8%)	
Lenvatinib or sunitinib	4 (7.7%)	0 (0%)	4 (11.1%)	

Abbreviation: ECOG PS, Eastern Corporation Oncology Group performance status; NOS, not otherwise specified.

### Efficacy and survival analysis

**FIGURE 2:**
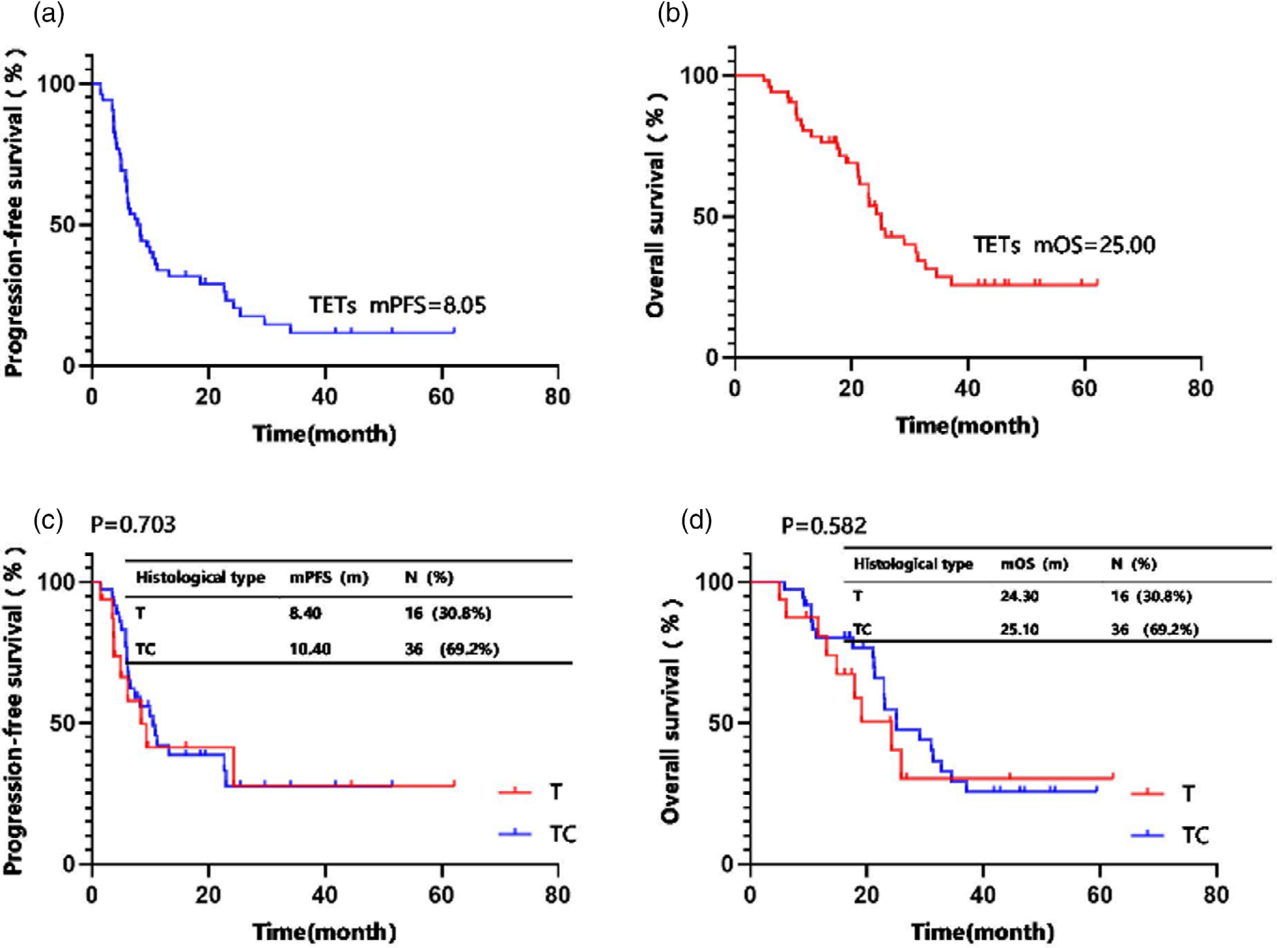
Kaplan–Meier analysis for progression‐free survival (PFS) and overall survival (OS) in all advanced thymic epithelial tumor (TET) patients (*n* = 52). (a) The median PFS and (b) median OS of patients with TETs were 8.05 and 25.0 months, respectively. (c) The median PFS of thymoma (T) and thymic carcinoma (TC). (d) The median OS of T and TC.

Based on reverse Kaplan–Meier analysis, follow‐up ended on August 10, 2022, for a median follow‐up period of 52.3 months. Among the 52 patients with TETs who received small molecule multitarget antiangiogenic drugs, none achieved CR, 21.1% (*n* = 11) showed PR, 73.1% (*n* = 38) showed SD, and 21.1% (*n* = 11) had PD (Table 2). The ORR was 21.1%, the DCR was 94.2% (Table 2), the mPFS was 8.05 months (Figure 2a), and the mOS was 25.00 months (Figure 2b). No CR was achieved in 16 patients with thymoma, 25.0% (*n* = 4) achieved PR, 56.3% (*n* = 9) showed SD, and 18.7% (*n* = 3) had PD, resulting in ORR and DCR of 25.0% and 81.3%, respectively (Table 2). The mPFS was 8.40 months (Figure 2c), and the mOS was 24.30 months (Figure 2d). No CR or PD was achieved in 36 patients with thymic carcinoma, 19.4% (*n* = 7) obtained PR, and 80% (*n* = 29) showed SD. In Table 2, their ORR and DCR correspond to 19.4% and 100.0%, respectively. In addition, the mPFS lasts 10.40 months as shown in Figure 2c, while the mOS reaches 25.10 months as shown in Figure 2d. No statistical differences were observed in mPFS (8.40 vs. 10.40 months, *p* = 0.703) and mOS (24.30 vs. 25.10 months, *p* = 0.582) between the two groups (Figure 2c,d).

**TABLE 2:** Response to antiangiogenic drugs in patients with thymic epithelial tumors.

Best overall response	Total (*N* = 52)	Thymoma (*N* = 16)	Thymic carcinoma (*N* = 36)
PR	11 (21.1%)	4 (25.0%)	7 (19.4%)
SD	38 (73.1%)	9 (56.3%)	29 (80.6%)
PD	3 (5.8%)	3 (18.7%)	0 (0.0%)
ORR	11 (21.1%)	4 (25.0%)	7 (19.4%)
DCR	49 (94.2%)	13 (81.3%)	36 (100.0%)

Abbreviations: DCR, disease control rate; ORR, overall response rate; PD, progressive disease; PR, partial response; SD, stable disease.

**FIGURE 3:**
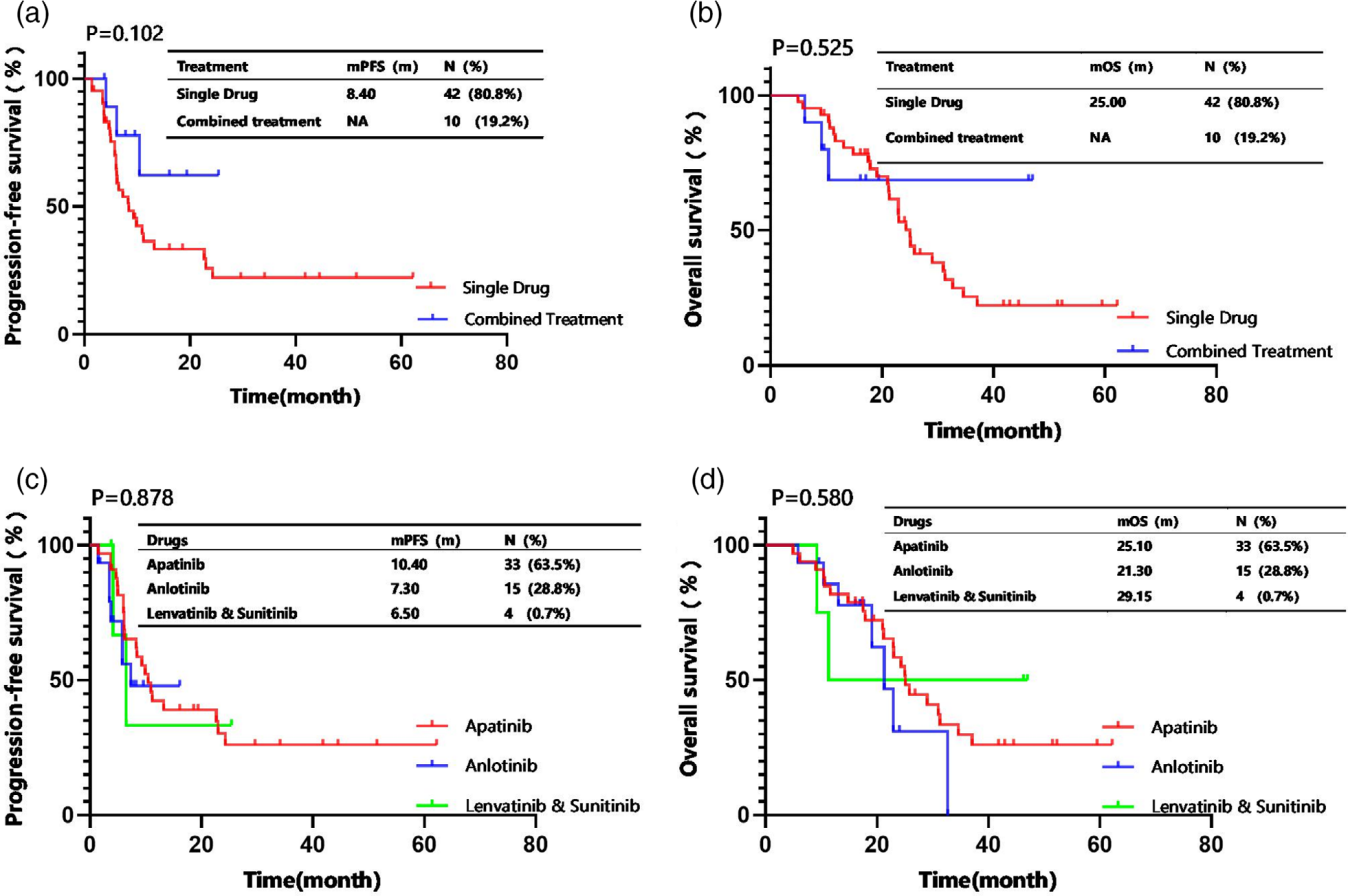
Kaplan–Meier analysis for (a) median progression‐free survival (PFS) and (b) median overall survival (OS) in patients with thymic epithelial tumors (TETs) treated with single drug or combined treatment. The median PFS (c) and the median OS (d) of patients with TETs treated with apatinib, anlotinib, lenvatinib, or sunitinib.

To further explore whether the effectiveness of single‐drug monotherapy (*n* = 42) was distinctive from combined therapy (*n* = 10) on these patients, we evaluated their mPFS (8.40 vs. not available, *p* = 0.102) (Figure 3a) and mOS (25.00 vs. not available, *p* = 0.525) (Figure 3b) but found no statistical differences. Meanwhile, no statistical differences were found in the mPFS (10.40 vs. 7.30 vs. 6.50 months, *p* = 0.878) (Figure 3c) and mOS (25.10 vs. 21.30 vs. 29.15 months, *p* = 0.580) (Figure 3d) among the patients treated with apatinib (*n* = 33), anlotinib (*n* = 15), and lenvatinib or sunitinib (*n* = 4).

### Prognostic analysis of patients with TETs

Univariate binary logistic regressions indicated that the combined treatment was associated with a superior OS (OR: 0.214, 95% CI: 0.048–0.957, *p* = 0.044) (Table 3). Additionally, multivariate binary logistic regressions further revealed that the combined treatment was an independent prognostic factor for PFS (OR: 0.038, 95% CI: 0.003–0.484, *p* = 0.012) (Table 3) and OS (OR: 0.026, 95% CI: 0.001–0.475, *p* = 0.014) (Table 3).

**TABLE 3:** Univariate and multivariate binary logistic regression analysis of baseline characteristics of patients with TETs associated with progression‐free survival (a) and overall survival (b).

Factor	Univariate analysis			Multivariate analysis		
	OR	95% CI	*p*‐value	OR	95% CI	*p*‐value
(a) Progression‐free survival						
Age (<60; ≥60)	2.000	0.3743–10.700	0.418	1.864	0.218–15.934	0.569
Gender (male; female)	1.333	0.335–5.311	0.683	<0.001	NA	1.000
Smoking history (positive; negative)	1.471	0.368–5.870	0.585	>99	NA	1.000
ECOG PS (0–1;2)	0.692	0.064–7.453	0.762	0.419	0.016–10.657	0.598
Masaoka‐Koga staging (IVA; IVB)	0.822	0.085–7.937	0.866	0.223	0.005–9.644	0.435
Histological type (T; TC)	1.667	0.398–6.974	0.484	1.364	0.135–13.755	0.792
Liver metastases (positive; negative)	0.672	0.162–2.794	0.585	0.518	0.063–4.290	0.542
Number of metastatic lesions (1; ≥2)	2.571	0.517–12.801	0.249	2.673	0.266–26.909	0.404
Surgical operation history (positive; negative)	0.551	0.135–2.241	0.405	0.185	0.015–2.325	0.191
Radiotherapy history (positive; negative)	2.143	0.443–10.375	0.344	6.204	0.572–67.349	0.134
Prior therapy lines (0–1; ≥2)	0.750	0.182–3.098	0.691	0.565	0.081–3.940	0.564
Antiangiogenesis therapy (single drug; combined treatment)	0.250	0.054–1.157	0.076	0.038	0.003–0.484	0.012
Antiangiogenesis drugs (apatinib, anlotinib, lenvatinib, or sunitinib)	1.144	0.371–3.526	0.814	2.189	0.355–13.498	0.398
(b) Overall survival						
Age (<60; ≥60)	1.760	0.507–6.112	0.373	2.106	0.387–11.456	0.389
Gender (male; female)	0.656	0.213–2.027	0.464	<0.001	NA	1.000
Smoking history (positive; negative)	0.747	0.241–2.312	0.613	>99	NA	1.000
ECOG PS (0–1;2)	2.143	0.207–22.130	0.522	1.323	0.056–31.486	0.863
Masaoka‐Koga staging (IVA; IVB)	0.711	0.118–4.281	0.709	0.254	0.010–6.459	0.407
Histological type (T; TC)	1.222	0.371–4.032	0.742	0.780	0.127–4.798	0.789
Liver metastases (positive; negative)	0.952	0.293–3.097	0.935	0.414	0.066–2.596	0.347
Number of metastatic lesions (1; ≥2)	0.694	0.153–3.152	0.637	0.426	0.052–3.474	0.426
Surgical operation history (positive; negative)	0.389	0.124–1.214	0.104	0.149	0.018–1.229	0.077
Radiotherapy history (positive; negative)	0.571	0.130–2.521	0.460	1.196	0.161–8.893	0.861
Prior therapy lines (0–1; ≥2)	1.100	0.342–3.537	0.873	1.130	0.237–5.385	0.879
Antiangiogenesis therapy (single drug; combined treatment)	0.214	0.048–0.957	0.044	0.026	0.001–0.475	0.014
Antiangiogenesis drugs (apatinib, anlotinib, lenvatinib or sunitinib)	0.584	0.242–1.412	0.233	0.828	0.233–2.948	0.771

Abbreviations: CI, confidence interval; ECOG PS, Eastern Corporation Oncology Group performance status; NA, not available; OR, odds ratio; T, thymoma; TC, thymic carcinoma; TETs, thymic epithelial tumors.

### Significant response to apatinib in patients with advanced thymic carcinoma and liver metastasis

**FIGURE 4:**
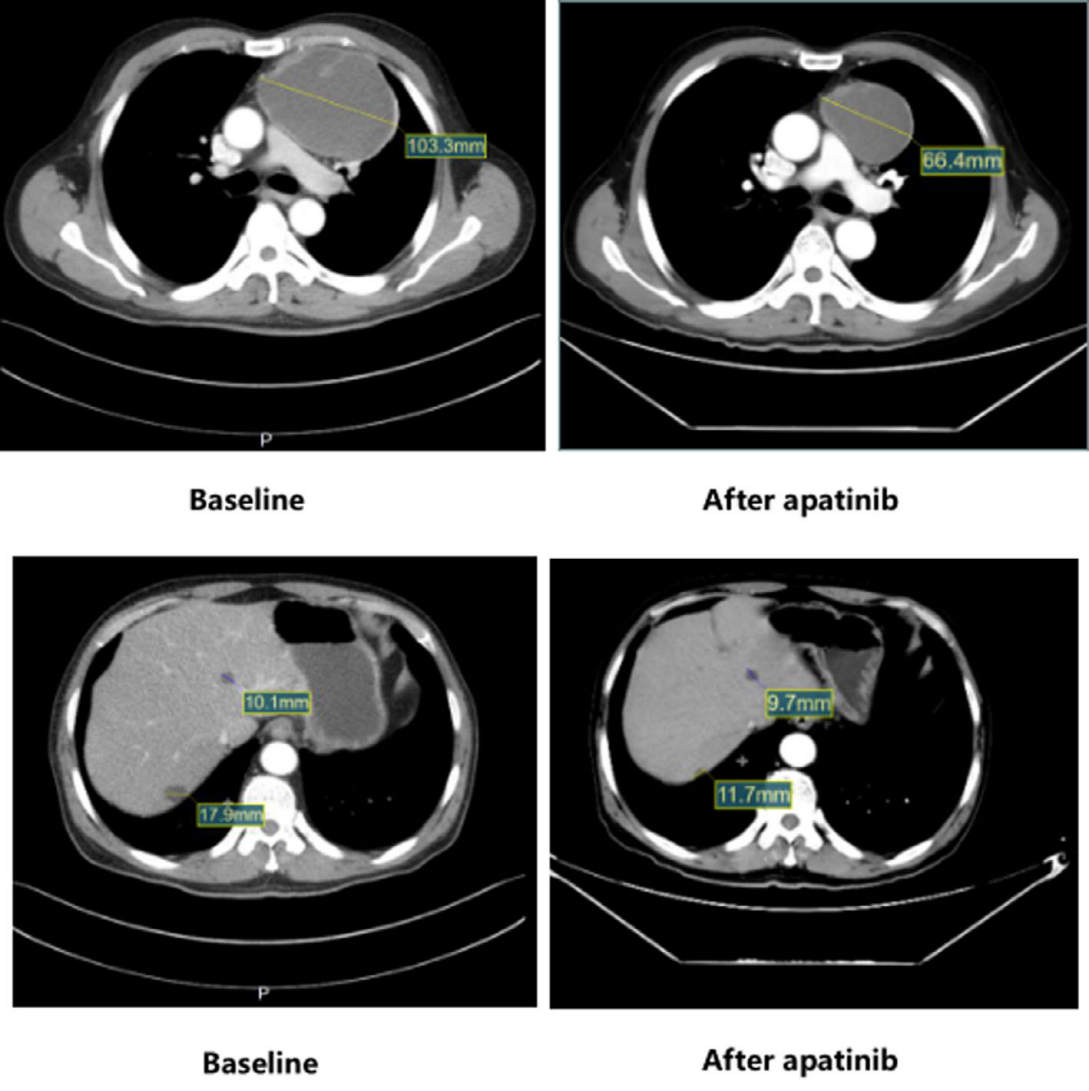
Representative pretreatment enhanced computed tomography images (left row) and post‐treatment images (right row) are shown for a 63‐year‐old man diagnosed with stage IVB thymic carcinoma with liver metastases. After 19 months of second‐line apatinib single‐agent treatment, the size of the primary lesion was significantly reduced, while the liver metastases remained stable.

A 63‐year‐old male was diagnosed with IVB (Masaoka‐Koga staging) thymic carcinoma with liver metastasis. He received two cycles of platinum‐containing chemotherapy as the first‐line treatment from January 17, 2019 to February 11, 2019. After two cycles of treatment at Zhejiang Provincial Cancer Hospital, the patient experienced disease progression in the liver metastasis. Then, he received apatinib monotherapy as the second‐line treatment from March 5, 2019, later confirmed as a partial response by computed tomography scanning. His PFS was 41.80 months, the primary lesion was significantly reduced, and the liver metastases remained stable (Figure 4).

## DISCUSSION

As TET is a relatively rare and indolent tumor, the patients can survive a considerable length of time after disease progression or relapse, making it extremely difficult to conduct large‐scale prospective randomized studies.^19^ To the best of our knowledge, this real‐world retrospective study of small molecule multitargeted antiangiogenic inhibitors on TETs in China involved a considerable sample size compared with other recent studies (Table 4). Our analysis showed a durable response, indicated by the ORR of 21.1%, the mPFS of 8.05 months (Figure 2a), and the mOS of 25.00 months (Figure 2b). No statistical differences in the mPFS or mOS were found between the 16 patients with thymoma and 36 patients with thymic carcinoma (Figure 2c,d). Similarly, a single‐arm phase II clinical trial in China included 25 patients with advanced TETs who failed platinum‐containing chemotherapy, were given 500 mg orally apatinib per day, and reported an overall population ORR of 40.0%, mPFS of 9.00 months, and mOS of 24.00 months, indicating the antitumor activity of the treatment.^14^ Meanwhile, Proto et al.^17^ conducted a multicenter Simon 2‐stage phase II trial, and reported that sunitinib as a second‐line treatment achieved an ORR of 21.7% at stage 2 in 32 patients with advanced or recurrent thymic carcinoma.

**TABLE 4:** Small molecule antiangiogenic therapies in TETs.

Study	Patients (T, TC)	Drug	ORR	mPFS (months)	mOS (months)
Phase II, Thomas et al.^11^	41 (16;25)	Sunitinib	T:6.0%; TC:26.0%	T:8.50; TC:7.20	T:15.50; TC:NA
Phase II, Sato et al.^12^	42 (−;42)	Lenvatinib	38.0%	9.30	NA
Phase II, Perrino et al.^13^	19 (11; 8)	Regorafenib	5.3%	9.60	33.80
Phase II, Song et al.^14^	25 (10;15)	Apatinib	T:70.0%; TC:20.0%	T:9.50; TC:6.10	T:22.40; TC:24.00
Retrospective, Antonarelli et al.^15^	20 (8;12)	Sunitinib	31.6%	7.30	NA
Phase II, Proto et al.^17^	44 (12;32)	Sunitinib	T: 0.0%; TC:21.7%	T:7.70; TC:8.80	T:47.90; TC:27.80
Phase II, Conforti et al.^18^	32 (5;27)	Avelumab plus axitinib	34.0%	7.50	26.6

Abbreviations: mOS, median overall survival; mPFS, median progression‐free survival; NA, not available; ORR, overall response rate; T, thymoma; TC, thymic carcinoma; TETs, thymic epithelial tumors.

In the current study, the mPFS (Figure 3a) and mOS (Figure 3b) of the single‐drug monotherapy group were 8.40 and 25.00 months, respectively, while the mPFS (Figure 3a) and mOS (Figure 3b) of the combined therapy group were not available. Multivariate binary logistic regressions revealed that combined treatment was an independent prognostic factor for PFS and OS (Table 3), suggesting that the combination treatment regime might be a potentially effective predictor for the prognosis of patients with advanced TETs. Among the combined therapy group, the majority of patients received immune checkpoint inhibitors as the combined therapy drugs. Previous studies have reported the antitumor activity of second‐ or post‐line pembrolizumab. Cho et al. conducted a single‐arm phase II study including 33 patients with TETs with previous chemotherapy failure; the ORRs of the patients with thymoma and thymic carcinoma were 28.6% and 19.2%, respectively, and the mPFS was 6.10 months for both.^20^ Another phase II study on pembrolizumab in 40 patients with advanced thymic carcinoma reported an ORR of 22.5%.^21^ Additionally, in another study, the incidence of grade 3–5 immune‐related adverse reactions was quite high; 26.4% in TETs, 58.3% in thymoma, and 17.1% in thymic carcinoma, which requires close monitoring.^22^ Angiogenesis is necessary for the genesis of TETs, and the architectural changes related to angiogenesis can hamper immune trafficking and immune cell metabolism, thereby reducing antitumor activities.^23^ Antiangiogenesis drugs can enhance the efficacy of immunotherapy.^24^ Conforti et al. conducted a single‐arm multicenter phase II study to evaluate the efficacy of avelumab plus axitinib combined therapy in 32 patients with advanced TETs, and found an overall ORR of 34%, an mPFS of 7.50 months, and an mOS of 26.6 months.^18^ Another phase II study on the effect of combined pembrolizumab and lenvatinib (NCT04710628)^25^ on patients with advanced TETs is ongoing.

Our study showed that the mPFS and mOS of the patients treated with apatinib were 10.40 and 25.10 months, anlotinib were 7.30 and 21.30 months, and lenvatinib or sunitinib were 6.50 and 29.15 months. Apatinib is a highly selective inhibitor of VEGFR2 and anlotinib is a VEGFR2/3 inhibitor, the treatment of which previously achieved ORRs of 40% and 9.1%, respectively, in patients with advanced TETs.^14^, ^16^ As promising new drugs for thymic carcinoma, angiogenesis inhibitors such as sunitinib and lenvatinib previously achieved ORRs of 26.0% and 38.0%, respectively.^26^

There were certain limitations in this study. First, the sample size of TETs patients was small due to the fact that TETs are relatively rare. Second, our study was single‐center and retrospective. Finally, data of adverse events were not reported due to the lack of complete records.

In conclusion, small molecule multitarget antiangiogenic inhibitors possess certain efficacy in treating patients with advanced TETs and may serve as an alternative therapeutic option for second‐ and further‐line treatment for patients with advanced TETs. Furthermore, the effect of combined small molecule antiangiogenic inhibitors treatment and chemotherapy/immunotherapy needs to be further explored in the future.

## AUTHOR CONTRIBUTIONS

Y.F. conceived the idea and designed the framework of the manuscript. W.S. conducted data analysis and interpretation, performed literature searches, and wrote the manuscript. Y.J. revised the entire manuscript and ensured its integrity. Y.Y. and N.C. contributed to data acquisition. All authors have read and agreed to the published version of the manuscript.

## CONFLICT OF INTEREST STATEMENT

The authors declare no conflicts of interest.
